# Sustained reduction of catheter-associated bloodstream infections by simulator-training and self-assessment

**DOI:** 10.1186/1753-6561-5-S6-O13

**Published:** 2011-06-29

**Authors:** W Zingg, V Cartier, C Inan, S Touveneau, F Clergue, D Pittet, B Walder

**Affiliations:** 1 Infection control programme, University of Geneva Hospitals, Geneva, Switzerland; 2 Division of Anaesthesiology, University of Geneva Hospitals, Geneva, Switzerland

## Introduction / objectives

Central line-associated bloodstream infection (CLABSI) is an avoidable complication in central venous catheter (CVC) use.

## Methods

In this study at the University of Geneva Hospitals, individual CVCs were prospectively observed hospital-wide in all adult patients. A baseline period (9/2006-12/2006) was followed by an intervention (1/2008-12/2008) and a sustainability period (1/2009-12/2009). Primary outcome was CLABSI. Interventions aimed at catheter insertion by anaesthesiologists and included (1) a comprehensive checklist, (2) a ready-to-use CVC-insertion set, (3) a CVC-insertion cart containing all necessary material, (4) self-assessment of insertion practice using online documentation, (5) simulation-based CVC-insertion training for residents, (6) web-based information site and (7) feedback during postgraduate education. No intervention was done outside the anaesthesiology division.

## Results

Anaesthesiologists, intensivists and other physicians placed 1665 (42%), 1693 (43%), and 617 (15%) catheters, respectively. Cumulative catheter-days and median (IQR) dwell-time were 35,914 and 6 (3-11) days, respectively. Most CVCs were jugular (62%), followed by subclavian (23%) and femoral (15%). CLABSI-rates of anaesthesiologists, intensivists and others at baseline, intervention and sustainability were 4.9, 2.9, 2.0 (IRR 0.75; 95%CI 0.57-0.99; p=0.04); 2.7, 1.4, 2.2 (0.96; 95%CI 0.63-1.46; p=0.85); and 1.6, 2.1, 3.9 (1.54; 95%CI 0.83-2.84; p=0.17), respectively.

## Conclusion

Improving CVC-insertion results in significant and sustained CLABSI-reduction. We consider self-assessment at catheter insertion and simulation-based training to have contributed most to the success.

## Disclosure of interest

None declared.

